# A Retrospective Analysis Assessing Paravalvular Leak and Pacemaker Implantation Using TEE and Non‐Contrast CT for CKD Patients Compared With CT Angiography for Annular Sizing Pre‐TAVR

**DOI:** 10.1002/hsr2.70847

**Published:** 2025-05-22

**Authors:** Michael O'Shaughnessy, Roxana Tabrizi, Derek Pham, Nicholas Jackson, Olcay Aksoy, Andre Akhondi, Jeanne Huchting, Richard Shemin, Murray Kwon, Peyman Benharash, Brandon Kim, Amir Rabbani

**Affiliations:** ^1^ Department of Medicine David Geffen School of Medicine Los Angeles California USA; ^2^ Department of Medicine, Division of General Internal Medicine and Health Services Research David Geffen School of Medicine Los Angeles California USA; ^3^ Department of Medicine, Division of Cardiology David Geffen School of Medicine Los Angeles California USA; ^4^ Department of Surgery, Division of Cardiac Surgery David Geffen School of Medicine Los Angeles California USA; ^5^ University of California Los Angeles California USA

**Keywords:** aortic stenosis, pacemaker implantation, paravalvular leak, TAVR, transcatheter aortic valve replacement

## Abstract

**Background and Aims:**

Transcatheter aortic valve replacement (TAVR) has become the treatment of choice for many patients with severe aortic stenosis. Proper pre‐procedure sizing of the aortic annulus is crucial in preventing post‐TAVR complications. This is typically performed with CT angiography, but the use of contrast is controversial in patients with chronic kidney disease (CKD).

**Methods:**

This study of 557 patients from 2016 to 2021 sought to evaluate a contrast‐sparing protocol for balloon expandable TAVR evaluation in patients with CKD, in which patients with glomerular filtration rate of less than 40 would undergo transesophageal echocardiogram (TEE) and CT without contrast (83 patients) for aortic annular sizing instead of CT angiography (445 patients).

**Results:**

We found that there was no significant difference in rates of greater than trace or greater than mild paravalvular leak between the two groups at hospital discharge, 30 days, or 1‐year post‐TAVR. We also found no difference in rates of permanent pacemaker implantation at these same time points.

**Conclusions:**

This suggests that TEE and non‐contrast CT could be a viable alternative to CTA in patients with CKD, although more research into other variables such as mortality and other post‐procedural complications is necessary.

## Summary

- We performed a retrospective cohort study on over 500 TAVR recipients between 2016 and 2021 to compare rates of PVL and PPM implantation at up to 1 year postprocedure between groups who underwent TEE and non‐contrast CT and those who underwent CTA. We found no significant difference in incidence of PVL or PPM implantation between TEE/CT non‐contrast and CTA at discharge, 30 days, or 1 year, indicating that TEE/non‐contrast CT may be a viable alternative to CTA in CKD patients.

## 1 Introduction

Transcatheter aortic valve replacement (TAVR) has risen in prominence over the past decade, becoming the treatment of choice for the majority of patients with severe aortic stenosis. Proper pre‐procedure sizing of the aortic annulus is crucial in preventing post‐TAVR complications but is complicated by factors such as the elliptical shape of the annulus, understanding the burden of leaflet calcification, and accurate assessment of the coronary heights. CT angiography (CTA) has become the standard for aortic annular sizing, offering precise anatomical detail. Compared to other imaging modalities, CTA enhances annular border visibility and provides critical information for vascular access planning, which traditional non‐contrast CT lacks [1]. However, the use of intravenous (IV) contrast remains potentially problematic in patients with chronic kidney disease (CKD) due to the possible risk of contrast‐induced nephropathy. Patients with a glomerular filtration rate (GFR) of less than 30 mL/min/1.73 m^2^ are classified as high risk for contrast‐induced nephropathy, while patients with a GFR of 30–45 mL/min/1.73 m^2^ are classified as intermediate risk [2]. The selection of imaging modality is therefore impacted by patient‐specific factors.

Paravalvular leak (PVL) is a common and highly morbid complication of TAVR. Even mild paravalvular leak has been shown in large‐scale studies to increase risk of rehospitalization in patients who have undergone TAVR, as well as increase both all‐cause and cardiovascular mortality [3, 4, 5]. In addition to paravalvular leak, another well‐known complication post‐TAVR is the need for permanent pacemaker (PPM) implantation. A recent systematic review reported PPM implantation rates after TAVR with new‐generation valves ranging from 2.3% to 36.1%, with higher rates for the early Medtronic CoreValve (16.3%–37.7%) and its successor, the Evolut R (14.7%–26.7%), while the Edwards SAPIEN 3 showed a lower risk (4%–24%) [6]. As PPM placement can impact a patient's length of stay, procedural risks, and mortality, utilizing effective preprocedural imaging technique is essential to evaluate the anatomic variables that can increase the chance for PPM [7].

Recent studies have suggested that transesophageal echocardiogram (TEE) may provide similar annulus size data to CTA, but data is mixed; however, to our knowledge the use of TEE for annular sizing in patients with renal insufficiency has not been evaluated in large‐scale studies [8, 9, 10].

We sought to evaluate a contrast‐sparing protocol at our center, in which patients with severe chronic kidney disease (defined as patients with a GFR of less than 40 mL/min/1.73 m^2^) undergo TEE and non‐contrast CT for annular sizing instead of CTA. This study specifically sought to evaluate this protocol in terms of incidence of PVL and PPM implantation at up to 1 year post‐TAVR.

## 2 Methods

In this retrospective cohort study, we evaluated the differences in incidence of PVL on post‐procedural transthoracic echocardiogram (TTE) and rates of PPM implantation post‐TAVR in patients who underwent preprocedural CTA vs. TEE and non‐contrast CT for annulus sizing and vascular access, using a GFR of less than 40 mL/min/1.73 m^2^ as the cutoff for CTA. GFR was calculated using the CKD‐EPI GFR calculator. Patients were informed of the use of this protocol before evaluation. We utilized non‐contrast CT to evaluate aortic annular calcium burden and provide an approximate annular size estimate, while TEE was employed for more precise annular sizing; in cases of measurement discrepancies or borderline area values between prosthesis sizes, the final prosthesis size selection was determined by integrating the TEE‐derived annular dimensions with the non‐contrast CT calcium assessment, prioritizing the TEE measurements for accuracy. Non‐contrast CT and TEE were performed pre‐procedurally for annular sizing in the majority of cases, with transthoracic echocardiography (TTE) routinely used intraprocedurally to assess PVL during implantation, while TEE was employed intra‐operatively in rare instances.

Our study population included patients from a tertiary care center in Los Angeles who received TAVR with the Edwards SAPIEN 3 balloon expandable valve between January 1, 2016 and December 31, 2021. Patients with end‐stage renal disease on hemodialysis were excluded (*n* = 29). Typical preoperative risk stratification was performed, and patients with active infection or other serious barriers to operative intervention did not undergo the procedure. Data were obtained from the electronic medical record and an internal TAVR database containing patient‐specific baseline and follow‐up information.

Group differences in baseline characteristics, incidence of PVL and rates of PPM placement were compared using Fisher's exact test and Welch's Two Sample *t*‐test, appropriate to variable distribution. Paravalvular leak on postoperative TTE was compared in two analyses: PVL greater than trace and PVL greater than mild. Patients lost to follow‐up or deceased were included in the analysis until the time they were lost to follow‐up or the time of death. A *p*‐value of < 0.05 was considered significant. Statistical analyses were conducted using R Version 4.2.1 (RStudio Team 2020; Integrated Development for R. RStudio Inc., Boston, MA http://www.rstudio.com/).

## 3 Results

### 3.1 Description of Cohort

Baseline characteristics of the cohort are summarized in Table 1. A total of 557 patients underwent preprocedural imaging with either CTA (*n* = 445) or TEE combined with non‐contrast CT (*n* = 83). The mean age of patients in the CTA and TEE groups was 78.8 and 80.2 years, respectively. Both cohorts had a slight male predominance, and most patients self‐identified as white, with a small proportion identifying as Black or African American (2.5% in the CTA group and 3.6% in the TEE group).

**Table 1:** Baseline demographic, past medical, and cardiodiagnostic data of the two imaging modality groups. A *p*‐value of less than 0.05 was considered statistically significant.

Baseline demographics	CTA, *N* = 445^a^	TEE, *N* = 83^a^	*p*‐value^b^
Age at time of procedure	78.82 ± 11.02	80.19 ± 9.89	0.26
Sex			0.071
F	206 (46%)	29 (35%)	
* *M	239 (54%)	54 (65%)	
Race/ethnicity**			0.48
American Indian/Alaska Native	1 (0.2%)	0 (0%)	
Asian/Pacific Island	25 (5.6%)	7 (8.4%)	
Black	11 (2.5%)	3 (3.6%)	
Hispanic	56 (13%)	5 (6.0%)	
White	310 (70%)	62 (75%)	
Other	40 (9.0%)	6 (7.2%)	
Unknown	2 (0.4%)	0 (0%)	
BMI	27.24 ± 6.31	27.44 ± 7.25	0.81
History of prior PCI (percutaneous coronary intervention)	71 (16%)	25 (30%)	**0.005**
Prior CABG (coronary artery bypass graft)	45 (10%)	10 (12%)	0.56
Prior MI (myocardial infarction)	58 (13%)	16 (19%)	0.17
History of hypertension	414 (93%)	80 (96%)	0.33
History of diabetes	125 (28%)	33 (40%)	**0.037**
History of chronic lung disease	120 (27%)	17 (20%)	0.27
History of TIA or stroke	51 (11%)	12 (14%)	0.46
History of peripheral artery disease	142 (32%)	31 (37%)	0.37
Admission creatinine	0.96 ± 0.36	1.78 ± 0.66	**< 0.001**
Admission GFR	69.78 ± 16.43	35.46 ± 11.13	**< 0.001**
History of AFib/A flutter	147 (33%)	41 (49%)	**0.006**
STS Risk score	4.67 ± 3.50	7.18 ± 3.93	**< 0.001**
RBBB on EKG	52 (12%)	8 (9.6%)	0.71
1st degree AV block on EKG	47 (11%)	9 (11%)	> 0.99
Bifascicular block on EKG	18 (4.0%)	3 (3.6%)	> 0.99
Mean aortic valve gradient on TTE (mmHg)	38.95 ± 15.00	34.89 ± 14.03	**0.017**
Mean aortic valve area on TTE (cm^b^)	0.74 ± 0.21	0.74 ± 0.28	0.97
Aortic valve peak velocity on TTE (m/s)	3.89 ± 1.15	3.72 ± 0.73	0.086

*Note:* Bold values indicate statistical significance.

^a^ *n* (%); Mean ± SD.

^b^ Fisher's exact test; Welch Two Sample *t*‐test.

There were notable differences in comorbidities between the two groups. The TEE cohort had significantly higher rates of diabetes mellitus (40% vs. 28%, *p* = 0.037), prior percutaneous coronary intervention (30% vs. 16%, *p* = 0.005), and atrial fibrillation or flutter (49% vs. 33%, *p* = 0.006). Additionally, the TEE group had a lower mean left ventricular ejection fraction (52.69% vs. 57.39%, *p* = 0.011) and a higher proportion of patients with an ejection fraction < 40% (25% vs. 13%, *p* < 0.001). The TEE group also had a higher mean Society of Thoracic Surgeons (STS) risk score pre‐TAVR.

In the CTA group, 5.2% of patients had bicuspid aortic valves, compared to 3.6% in the TEE group. Follow‐up data were incomplete for 21% of CTA patients and 25% of TEE patients, with similar proportions of deceased patients at 1 year (9.7% vs. 9.9%, respectively). There were no significant differences between the two groups in electrocardiographic abnormalities, including right bundle branch block, first‐degree AV block, or bifascicular block.

### 3.2 Paravalvular Leak and Permanent Pacemaker Implantation

**Figure 1:**
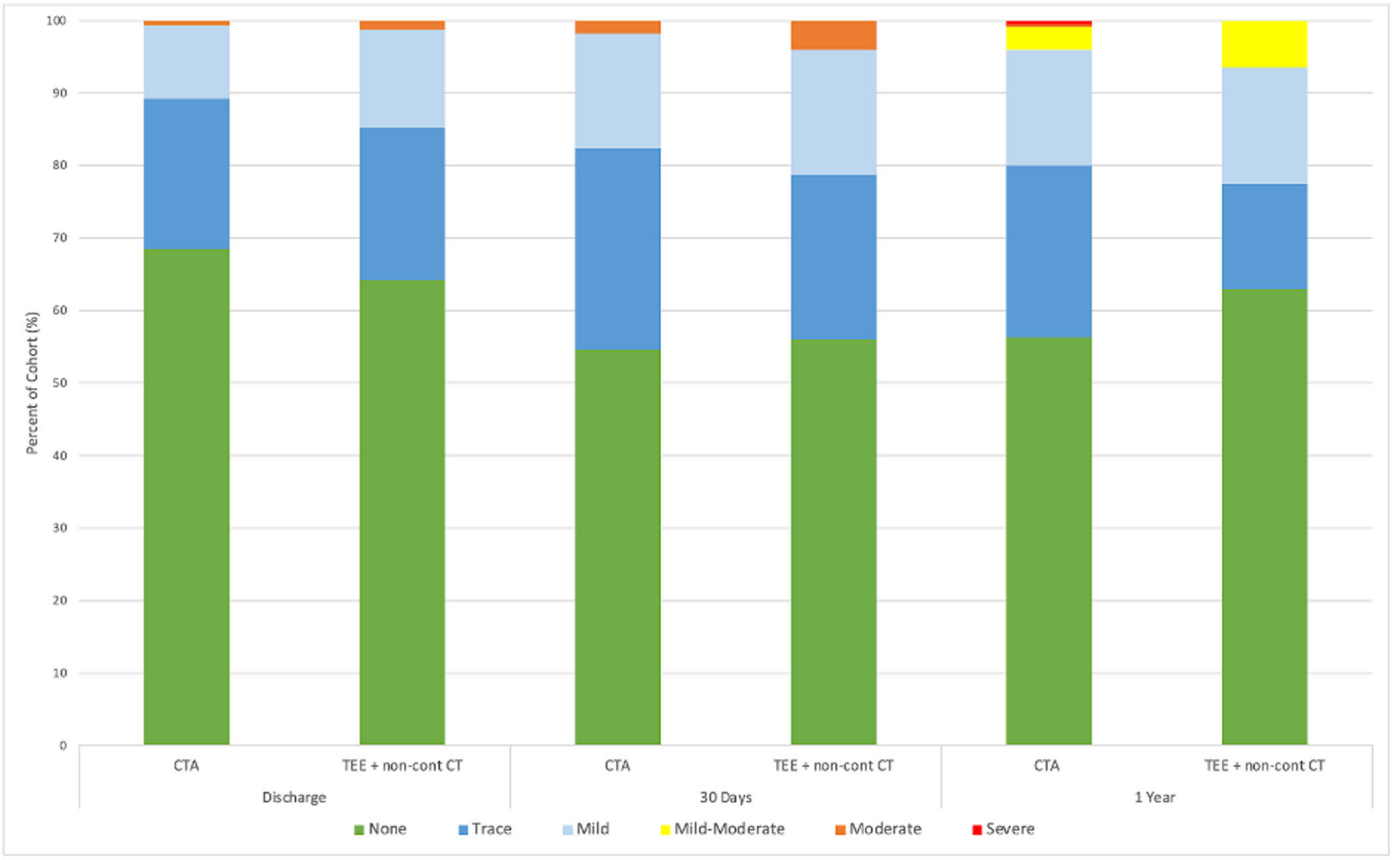
A visual representation of paravalvular leak severity in the two imaging modality groups at hospital discharge, 30 days postprocedure, and 1 year postprocedure.

A visual representation of paravalvular leak (PVL) severity across all follow‐up points is provided in Figure 1. The incidence of PVL greater than mild was low in both groups, with less than 10% of patients experiencing moderate or severe PVL at 1 year (6.5% in the CTA group vs. 4.0% in the TEE group). No statistically significant differences in PVL severity were observed between the groups at discharge, 30 days, or 1‐year post‐TAVR (Table 2).

**Table 2:** Comparison of both greater than trace and greater than mild paravalvular leak between the two imaging modality groups at discharge, 30 days, and 1 year post‐TAVR. A Fisher's exact test was used to compare the rates of PVL. *p*‐values less than 0.05 were considered statistically significant.

	Discharge		
	TEE + non‐cont CT	CTA	*p*‐value
PVL > Trace	14/83 (17%)	52/442 (12%)	0.21
PVL > Mild	3/83 (3.6%)	8/442 (1.8%)	0.39
	30 Days		
PVL > Trace	16/75 (21%)	78/395 (20%)	0.75
PVL > Mild	3/75 (4.0%)	17/395 (4.3%)	> 0.99
	1 Year		
PVL > Trace	14/62 (23%)	70/350 (20%)	0.61
PVL > Mild	4/62 (6.5%)	14/350 (4.0%)	0.33

**Figure 2:**
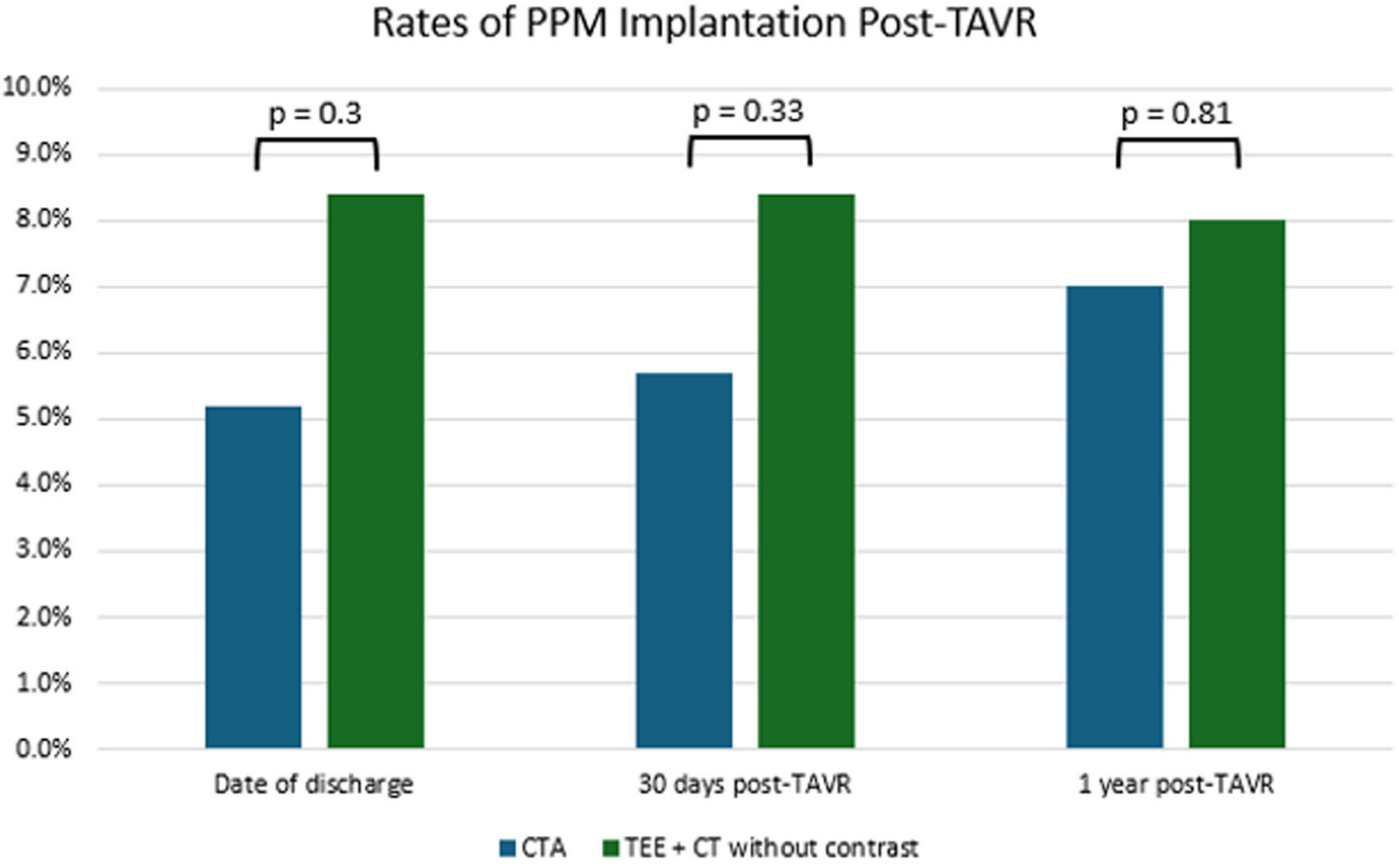
Rates of permanent pacemaker implantation at hospital discharge, 30 days, and 1 year post‐TAVR. Rates were compared using Fisher's exact test at a 95% confidence level.

Permanent pacemaker (PPM) implantation rates remained low at 1 year (7.0% in the CTA group vs. 8.0% in the TEE group), with no significant differences observed at discharge, 30 days, or 1‐year post‐TAVR (Figure 2).

## 4 Conclusions

In this retrospective cohort study, we examined the incidence of PVL and rates of PPM implantation following TAVR in patients who underwent preprocedural assessment with either CTA or TEE combined with non‐contrast CT. Our findings suggest that the choice between these imaging modalities does not significantly impact PVL incidence or the need for PPM implantation up to 1‐year postprocedure.

PVL, even when mild, has been associated with increased rates of rehospitalization, all‐cause and cardiovascular mortality, and worsening clinical outcomes. Nationally, PVL rates after TAVR vary widely due to factors such as operator experience, transcatheter valve type, imaging modality, and timing of assessment. Predictors of PVL include aortic root and valve leaflet calcification, pre‐TAVR transvalvular gradient, peak velocity across the aortic valve, and implantation depth [11, 12, 13, 14, 15].

While mild or trace PVL is common, occurring in an estimated 50%–70% of patients post‐TAVR, a meta‐analysis of 38 studies involving over 25,000 patients demonstrated a significant association between PVL and increased mortality risk (HR 1.52, 95% CI 1.43–1.61), rehospitalization (HR 1.81, 95% CI 1.54–2.12), and cardiovascular mortality (HR 1.52, 95% CI 1.33–1.75) [16]. Our results showed consistently low rates of PVL greater than mild across all follow‐up time points, with no meaningful differences between the CTA and TEE groups (Figure 1). These findings suggest that CTA does not confer substantial advantage over TEE in mitigating PVL when utilizing the Edwards SAPIEN 3 valve. The Edwards SAPIEN 3 valve design appears to effectively mitigate PVL regardless of the imaging modality used for preprocedural planning. Although CTA is expected to offer superior annular sizing and anatomical characterization, this did not translate into reduced PVL rates in our cohort.

PPM implantation following TAVR is most commonly indicated for high‐grade atrioventricular block, worsening or new‐onset left bundle branch block, progressive first‐degree AV block, pre‐existing right bundle branch block, unstable nodal conduction, or symptomatic bradycardia [17]. Given the anatomic relationship of the aortic valve to the bundle of His, this leads to vulnerability of the nearby conduction system, increasing the risk of PPM implantation. Contributing factors include implantation depth, membranous septum length, calcium burden in the noncoronary cusp, valve type, patient anatomy, and pre‐existing conduction abnormalities [18].

A prolonged QRS duration > 120 ms, regardless of conduction block type, is one of the strongest predictors of PPM implantation post‐TAVR, and diabetes mellitus has also been associated with higher PPM rates [19, 20]. Recent analysis has demonstrated that the median time of 3 days from TAVR to PPM implantation [21, 22]. Since PPM placement impacts hospital length of stay, procedural risk, and long‐term outcomes, optimizing preprocedural imaging is essential for identifying anatomical risk factors [23]. In our study, PPM implantation rates were similar between the CTA and TEE groups, with an overall low incidence at 1 year (7.0% vs. 8.0%, respectively). These findings likely reflect advancements in TAVR technology and procedural techniques, which have reduced conduction disturbances requiring PPM implantation [24, 25].

Despite baseline differences between the two groups—such as a higher prevalence of diabetes mellitus, prior PCI, and atrial fibrillation in the TEE group—no significant differences in PVL or PPM implantation rates were observed. This suggests that both imaging modalities provide reliable anatomical assessment and procedural guidance.

Our study has several limitations. The retrospective design and single‐center setting may introduce biases and limit generalizability. Additionally, the smaller sample size of the TEE cohort may reduce statistical power to detect subtle differences. We focused on clinically relevant endpoints—PVL and PPM implantation—which are less susceptible to confounding than mortality, given the differing risk profiles of the groups. Future research should validate these findings in larger, multicenter cohorts to improve robustness and generalizability. Prospective, randomized trials could further evaluate more comprehensive outcomes. Additionally, our results may not apply to self‐expanding valve systems, warranting further investigation in centers that predominantly use these devices.

In conclusion, our findings suggest that the choice of preprocedural imaging modality—CTA vs. TEE—does not significantly impact the incidence of PVL or necessity for PPM implantation in patients undergoing TAVR with the Edwards SAPIEN 3 valve. These findings support the flexibility in selecting an imaging modality based on patient‐specific factors and institutional preferences, without compromising clinical outcomes. Given the limitations of the study design, these findings should be considered hypothesis‐generating and serve as a basis for future research. Continued advancements in TAVR technology and procedural techniques are likely to further improve outcomes.

## Author Contributions

**Michael O'Shaughnessy:** conceptualization, data curation, formal analysis, methodology, writing – original draft, writing – review and editing. **Roxana Tabrizi:** data curation, formal analysis, writing – original draft, writing – review and editing. **Derek Pham:** formal analysis, software, writing – original draft, writing – review and editing. **Nicholas Jackson:** data curation, formal analysis, writing – original draft, writing – review and editing. **Olcay Aksoy:** conceptualization, formal analysis, writing – original draft, writing – review and editing. **Andre Akhondi:** conceptualization, writing – original draft, writing – review and editing. **Jeanne Huchting:** conceptualization, writing – original draft, writing – review and editing. **Richard Shemin:** conceptualization, writing – original draft, writing – review and editing. **Murray Kwon:** conceptualization, writing – original draft, writing – review and editing. **Peyman Benharash:** conceptualization, writing – original draft, writing – review and editing. **Brandon Kim:** data curation, writing – original draft, writing – review and editing. **Amir Rabbani:** conceptualization, data curation, formal analysis, project administration, supervision, writing – original draft, writing – review and editing.

## Disclosure

All authors have read and approved the final version of the manuscript; Michael O'Shaughnessy, MD and Amir Rabbani, MD had full access to all of the data in this study and take complete responsibility for the integrity of the data and the accuracy of the data analysis.

## Ethics Statement

Ethical approval for this study was granted by the UCLA Institutional Review Board (IRB # 16‐000680, “Treatment of Aortic Stenosis”). Informed consent was obtained from all patients before enrollment.

## Conflicts of Interest

The authors declare no conflicts of interest.

## Transparency Statement

The lead author Michael O'Shaughnessy affirms that this manuscript is an honest, accurate, and transparent account of the study being reported; that no important aspects of the study have been omitted; and that any discrepancies from the study as planned (and, if relevant, registered) have been explained.

## Data Availability

The data that support the findings of this study are available on request from the corresponding author, M.O.
